# Aesthetic injustice in healthcare: exploring testimonial and hermeneutical forms

**DOI:** 10.1007/s11019-026-10334-6

**Published:** 2026-03-03

**Authors:** Jonathan Adams

**Affiliations:** Centre for Medical Ethics, Institute of Health and Society, Faculty of Medicine, University of Oslo, Gaustadalléen 21, Forskningsparken, Hus C Plan 1, Oslo, 0349 Norway

**Keywords:** Aesthetic injustice, Epistemic injustice, Testimonial injustice, Hermeneutical injustice, Healthcare ethics, Body aesthetics

## Abstract

This paper introduces and develops the concepts of aesthetic testimonial injustice and aesthetic hermeneutical injustice, arguing that understanding the aesthetic dimensions of human experience, specifically those concerning physical appearance, is central to justice in healthcare practice. Drawing on Miranda Fricker’s framework of epistemic injustice, the paper provides a bridge between emerging literature on aesthetic injustice, which is often focused on lookism and appearance-based discrimination, and accounts that emphasize the recognition of individuals as aesthetic subjects. The concept of aesthetic testimonial injustice relates to the unjust discrediting of a person’s aesthetic testimony regarding their own body, often due to biases related to attractiveness, age or disability. Aesthetic hermeneutical injustice, by contrast, pertains to the structural disadvantage faced by individuals who lack the interpretive resources to make sense of or communicate their aesthetic experience as it pertains to bodily appearance, particularly in healthcare contexts. Using examples from prosthetics design, nursing care and disorders of visual perception, the paper shows how healthcare systems can marginalize patients’ aesthetic values and perceptions, thereby undermining their dignity and agency. It concludes by calling for a deeper understanding of aesthetics in bioethics, suggesting that respecting patients as embodied aesthetic agents can enhance ethical healthcare delivery and counter systemic injustice. The concepts of aesthetic testimonial injustice and aesthetic hermeneutical injustice thus expand bioethical discourse by highlighting the moral significance of aesthetic experience in healthcare contexts.

## Introduction

In recent philosophical work, there has been significant interest in analyzing aesthetic judgements and motivations through the lens of justice. Among practical ethicists, this work has focused on how a person may be subject to injustice due to the aesthetic judgements of another, typically those made about the recipient of injustice’s physical appearance. Referred to by such scholars as “lookism” (Mason 2021), “appearance discrimination” (Cavico et al. 2012), or “aesthetic injustice” (Hofmann 2024), this phenomenon occurs when a person is wronged because they are seen as unattractive.

Within another strand of philosophical work, however, scholars tend to discuss how a person may be subject to injustice as an aesthetic appraiser or creator, focusing therefore on the recipient of injustice’s creation, appraisal, and appreciation of works of art (Dalaqua 2020; Fraser 2024; Nanay 2024; Roberts 2025). With credibility, recognition, and understanding among its key ideals, this literature intersects naturally with the literature on epistemic injustice, which is concerned with how the recognition and receipt of knowledge can be fair or unfair.

In this paper, I will build a bridge between these two literatures by adopting a focus on a person’s physical appearance, like the first, and on the recipient of injustice as a subject of aesthetic judgement and experience, like the second. After some background on the concept of ‘aesthetic injustice’ that I will seek to explore and expand, the main body of the paper will delineate two novel forms of aesthetic injustice, drawing on Fricker’s (2007) seminal discussion of epistemic injustice. These forms are ‘aesthetic testimonial injustice’, involving dismissal of an individual’s testimony about how things ought aesthetically to be, and ‘aesthetic hermeneutical injustice’, involving the diminished capacity to grasp aesthetic judgement as a facet of social experience.

Given that this paper is apparently the first to introduce these novel forms of aesthetic injustice, its scope is necessarily narrowed to allow for detailed exposition, with the focus being specifically on aesthetic judgements that relate to human physical appearance. Aesthetic injustice could, in principle, relate to testimonies and understandings concerning any of the phenomena that Cribb and Pullin (2022) link to healthcare quality improvement discourse, ranging from physical environments to “forms and styles of communication” (p. 485). However, for the sake of simplicity, these issues are reserved for future work, and while more general definitions may be required in future, aesthetic testimonial injustice and aesthetic hermeneutical injustice are defined here explicitly with reference to bodily aesthetic judgements.

For each form of injustice, this paper will analyze how the practice of healthcare, which is deeply invested in human physical appearance and the social norms that concern it, can be seen as perpetuating the injustice. I will apply examples from the arts and health(care), but the phenomena are general and may be explored and elaborated upon in other areas. Examples from the arts will be used throughout not to suggest a special affinity between art and healthcare but because they offer rich illustrations of how aesthetic judgements operate that help to clarify similar patterns in clinical and caregiving contexts. In the discussion section, the significance and viability of aesthetic testimonial injustice and aesthetic hermeneutical injustice as concepts will be further analyzed, with a particular focus on how they build on prior recognition of the significance of aesthetic understanding in the field of bioethics, before the paper concludes.

## Background

Before exploring this paper’s novel approach, it is worth laying out some of the emerging philosophical approaches to human physical appearance, particularly insofar as they concern the ethical ideal and principle of justice.

This paper embraces the concept of 'aesthetic injustice', which is designed as an umbrella term encompassing, inter alia, what other scholars have called “lookism, heightism, sizeism, appearance discrimination, and beauty bias” (Hofmann 2024, p. 220). Of these individual concepts, scholarship in practical ethics has given particular attention to lookism and appearance discrimination, which tend to be dealt with as a single phenomenon (Liu 2017). For example, Widdows (2017) examines lookism in relation to the costs of compliance with beauty standards, while Mason (2021) argues that everyday lookism may be not only unfairly costly but also demeaning and threatening to personal autonomy. “Anti-lookist measures,” according to Minerva (2017, p. 5), could consist of “anti-discriminatory laws, affirmative action, and equal opportunities policies” and could be achieved, at least in some jurisdictions, by recognizing appearance as a protected characteristic under existing legislation (Mason and Minerva 2020).

When it comes to the application of the term ‘aesthetic’, some scholars may want to emphasize that the source of lookist discrimination need not itself be an aesthetic value judgement. Mason (2023) distinguishes between four possible causes of direct appearance discrimination, of which only one is a judgement about the aesthetic value of a particular feature in terms of its ‘ugliness’ or ‘unsightliness’, for example. Discrimination may instead come from “*prejudices or stereotypical associations*, for example, the association of good looks with greater competence,” “*non-rational responses* […] for which the agent has no motivating belief or reason,” or “*moral judgements* that express approval or disapproval of someone’s appearance” (Mason 2023, p. 10).

However, the notion that the injustice in such cases is ‘aesthetic’ allows for the factors involved to be complicated and suggests only that, whatever the source of appearance-based discrimination, it will somehow contain an aesthetic evaluation. As Tietje & Cresap (2005, p. 38) conceive of lookism, it is “primarily an aesthetic experience, an immediate attraction or repulsion at the physical presence of others,” even though it may be bound up with other social and psychological mechanisms. Mason is therefore right to identify proximate causes of appearance-based discrimination beyond just aesthetic valuation, yet they should be understood within a more complex web of factors that necessarily involves the aesthetic valuation of some appearances over others (Kuipers et al. 2019).

Two forms of injustice that are not typically identified in literature on lookism and appearance-based discrimination but have been included under the concept of aesthetic injustice are testimonial and hermeneutical injustices, as first conceptualized by Fricker (2007). Specifically, it has been suggested that “[p]eople who are considered to be unattractive are *less listened to* (*testimonial* injustice) and less trusted” and they “contribute less to concept formation and concept promotion (*hermeneutical injustice*)” (Hofmann 2024, p. 221).

Hofmann’s application of Fricker’s (2007, p. 1) concept of testimonial injustice, which “occurs when prejudice causes a hearer to give a deflated level of credibility to a speaker’s word,” is supported by studies suggesting that the testimony of attractive informants is likelier to be endorsed by hearers (Bascandziev and Harris 2016). To identify a “hermeneutical injustice,” which occurs “when a gap in collective interpretive resources puts someone at an unfair disadvantage when it comes to making sense of their social experiences” (Fricker 2007, p. 1), Hofmann cites Spiegel (2022). Spiegel states that prejudice against those deemed unattractive is unfairly rendered obscure to them by “the taboo of ugliness [that] causes ugly people to not fully appreciate the reasons for some of their ills” (2022, p. 54), which is potentially well-intentioned but, as a form of stigmatization, unjust.

Beyond these arguments, little has been said about the relationship between aesthetic and epistemic injustice, which seems to represent a missed opportunity given the large bodies of ethical theory on both subjects that now exist, and the importance of these types of injustice. In this paper, I will therefore look specifically at forms of injustice where there are no outside judgements made about the recipient of injustice’s appearance and, instead, it is the recipient whose aesthetic beliefs and understanding are of immediate relevance to the injustice. A consequence of this shift away from existing work on lookism and appearance-based discrimination is an underlying recognition that aesthetic injustice need not involve the deliberate wrongdoing of individual agents, nor even explicit aesthetic judgements. As this paper will explore, the kinds of disadvantage relevant to aesthetic testimonial injustice and aesthetic hermeneutical injustice may be due not to individual prejudice but to certain aesthetic perceptions occupying structurally advantaged or disadvantaged positions. This picture of aesthetic injustice may not provide a direct mapping of Fricker’s conception of epistemic injustice onto the aesthetic domain; however, it is consonant with recent accounts of epistemic injustice that build on Fricker’s original notion to emphasize its structural dimensions (Doan 2018; Reibold 2025).

## Aesthetic testimonial injustice in the arts and health/care

The concept of aesthetic testimonial injustice is novel but can, for present purposes, be defined as follows:

*Aesthetic testimonial injustice* – When a person’s testimony regarding aesthetic matters related to their own body is unjustly given less credibility due to prejudice, often linked to biases about their perceived competence or rationality.

The use of “testimonial” here refers exclusively to a person’s *expressed aesthetic self-understanding*, i.e. what they say about how their body ought to look or be aesthetically experienced, and not to others’ perceptions of their physical appearance. It is therefore important to distinguish between aesthetic testimonial injustice so defined, where a subject’s aesthetic testimony is dismissed, and other cases where any testimony is dismissed because of appearance. For example, obese patients often report that their health concerns are attributed solely to weight and their insights overlooked due to physicians’ appearance-based judgements (Lazris et al. 2023). These broader forms of appearance-related testimonial injustice are highly relevant in healthcare but deserve further study *in addition* to the phenomenon of aesthetic testimonial injustice as I understand it.

While there is little discussion in philosophical literature of the kind of body-related testimonies described here, the general notion of aesthetic testimony is well-known in discussions of the assertions that people typically make about artworks (Robson and Wallbank 2024). Thus, philosophers vigorously debate whether the testimony of others can provide knowledge about whether an artwork is beautiful or whether one requires experience of an artwork (Robson 2022).

In our everyday judgements about artworks, we certainly seem to put trust in the recommendations of friends, family, and art critics, even if it is only provisional (Hills 2020), and if we do so due to our prejudices, we may be guilty of testimonial injustice. Support for this view can be drawn from Fraser (2024), who argues that an aesthetic injustice occurs when, for example, women’s *artworks* (and not aesthetic judgements per se) are appraised more negatively than those of men. Fraser quotes the American artist Judy Chicago: “If it’s done by a man it’s high art; if it’s done by a woman it’s decorative” (Fraser 2024, p. 449).

Nanay (2024) takes the view that aesthetic assessment is an everyday feature of our experience and suggests, quite radically, that “we are all in danger of being culpable of aesthetic injustice on a daily or maybe even hourly basis” (p. 159). Specifically, Nanay locates an aesthetic injustice in the judgements made about artworks from other cultures, which involves “feeling more of an expert in someone else’s experience than that person” (2024, p. 158). However, in some ways, the body is a more natural locus than artworks for everyday aesthetic assessments, which will qualify as aesthetic testimonies if expressed verbally.

In the healthcare domain, Cribb and Pullin (2022) have applied the idea of everyday aesthetics by following Saito’s account of the aesthetic as involving “any reactions we form towards the sensuous and/or design qualities of any object, phenomenon or activity” (2007, p. 9). Cribb and Pullin (2022, p. 480) note that discussion of aesthetics in healthcare quality and improvement “is often confined to specific areas of concern, such as […] healthcare architecture” and instead look broadly at healthcare encounters. Though they also analyze the design of patients’ surroundings and physical objects such as pills and ointments, the authors note that “direct work on and with bodies, including links with ‘body image’, is the area of healthcare most associated with the word ‘aesthetic’” (Cribb and Pullin 2022, p. 483).

The design of prostheses is a particularly challenging area, where the aesthetic and practical preferences of designers may come into conflict with those of the individuals for whom prostheses are being designed. Cribb and Pullin note that the aesthetic value of a prosthetic hand has historically been “equated with cosmetic adequacy and/or simulation of a ‘realistic look’” and that consequently, “[t]he demands of form and function—or aesthetic adequacy and technical adequacy—were often assumed to be in conflict” (Cribb and Pullin 2022, p. 483) Yet where this perception prevails, it neglects the diversity of perspectives held by people with limb differences, who may eschew “‘realism’ […] to embrace or play with making their differences overt in various ways including by invoking a ‘transhuman’ identity” or choosing to “naturalise and normalise” difference (Cribb and Pullin 2022, p. 483).

A contrasting and striking example of a wearer devising new functions to achieve aesthetic ends is Jordan Reeves, an American thirteen-year-old born with a limb difference who designed her own prosthetic arm to resemble a “glitter-shooting unicorn horn” (WGN News 2019). As well as describing the visual appeal of glitter and the mechanism of distributing it, which she refers to as “kind of magical” and “a pretty cool thing,” the teenager uses the language of difference to capture its aesthetic value: “It shows that I may be different, but I can do so much more” (WGN News 2019).

The problem of aesthetic testimonial injustice is that there are barriers to the claims of individuals like Reeves being taken seriously as instances of testimony that put forward a genuinely rival account of what constitutes an aesthetically appealing limb. Shew (2019) remarks of Reeves’ design that “[w]hen people can determine the meaning and joy of their own bodies, there is much more room for creativity and play.” However, it seems that the ‘playfulness’ that Shew sees as a positive could be used against disabled people’s aesthetic testimonies in a way that resembles the dismissal of women’s aesthetic affirmations as ‘sentimental’. Whatever the specific terms on which they are rejected, prospective wearers’ testimonies as to the beauty of an alternative prosthesis will have to compete with able-bodied designers’ assertions, whether implicit or explicit, that ‘they’ should look like ‘us’ (Moser 2000).

While the use of prostheses is quite rare, of course, other scholarship in the field of the aesthetics of everyday life points us towards instances of everyday aesthetic testimony that occur in the practice of care that we could all receive in our lifetimes. In particular, Pols’s work on “the aesthetic side of dignity” (2013a, p. 954) stresses the importance of aesthetic values, which consist in “different conventions of what is proper, tasteful, stylish, or pleasant” (2013a, p. 953) in care, including nursing care. Everyday aesthetic testimony is a necessary feature of such an area of practice, where the patient may be incapable of attending to their own appearance and rely on demands upon others to be dressed, bathed, and adorned in the ways that they regard to be proper.

A key area of aesthetic value on which conflicting aesthetic testimonies may be offered, and some of them unjustly rejected, is that of cleanliness and uncleanliness, which are pervasive ideas in everyday life but almost entirely ignored in philosophical aesthetics. Suggesting that they constitute a “class of neglected properties,” Leddy (1995, p. 259) assembles a list of aesthetic adjectives including ‘“neat,” “messy,” “clean/unclean,” “dirty,” “sloppy,” “filthy,” [and] “ordered/disordered”'. Thus, when a patient states that their hair looks ‘too messy’, their skin seems ‘too dirty’, or their clothes feel ‘too sloppy’, they express a view about their desired aesthetic qualities that those in control may or may not take seriously. Equally, patients might testify that their appearance is aesthetically pleasing, or at least sufficient, where caregivers consider it sloppy, as Pols (2013b, pp. 193–194) suggests: “Hardly ever did the patients themselves object to spots on their clothing or unwashed appearances […] However, family and carers *did* care.”

In the cases of expressed aesthetic preferences in social care and prosthesis design, it does not seem that caregivers or designers have an obligation to accept the aesthetic testimonies of patients or users but rather to take them seriously as expressions of a legitimate aesthetic sensibility. Thus, the just attribution of credibility is a matter of seeing a testifier not so much as a potential guide for one’s beliefs than as a guide for action based on the interpersonal knowledge revealed to us and the requirement to respect personal autonomy (Wiltsher 2024; Martin-Seaver 2023). Obstacles to testimonial justice may therefore be identified in prejudices that, for example, treat older people’s disregard for aesthetic maintenance as a form of self-neglect (Dahl et al. 2018) or suggest that visual impairment uniformly impedes aesthetic understanding (Feeney 2019).

## Aesthetic hermeneutical injustice in the arts and health/care

Let us define the concept of aesthetic hermeneutical injustice, such as it is relevant to the context of this paper, as below:

*Aesthetic hermeneutical injustice* – when individuals are unfairly disadvantaged in making sense of, communicating, or participating in shared practices involving aesthetic evaluation of the body.

The concept of aesthetic hermeneutical injustice therefore constitutes a new, domain-specific form of hermeneutical injustice, which on Fricker’s account concerns marginalization from shared interpretive resources in any epistemic domain. Specifically, the concept focuses on hermeneutical injustice that arises when individuals’ non-standard or atypical ways of aesthetically perceiving and experiencing the body are not recognized within dominant interpretive frameworks. In this sense, the phenomenon is at bottom hermeneutical: it reflects a structural disadvantage in making sense of and communicating bodily aesthetic experience and thus extends Fricker’s hermeneutical notion into the domain of bodily aesthetics. Similarly to aesthetic testimonial injustice, this exact phenomenon has not been described in existing literature, but there are philosophical reference points when it comes to the evaluation of works of art that can be applied to bodily aesthetics for our healthcare-oriented analysis. Notably, Dalaqua’s (2020) idea of aesthetic injustice, which he describes by analogy with epistemic injustice as “any harm done to someone specifically with regards to her aesthetic capacities” (p. 1), contains a substantial hermeneutical element.

Dalaqua argues that an aesthetic injustice occurs when the oppressed “become uninterested in and numb to the art and knowledge produced by their own people, which in turn arrests the development of their cognitive and aesthetic capacities” (2020, p. 2). Dalaqua identifies the subsequent numbing of persons’ awareness of their oppression as an epistemic injustice, yet this aesthetic numbing can itself be understood as a hermeneutical injustice, if aesthetic appreciation is taken as a kind of 'social experience’, in Fricker's sense.

There are also vast gaps observed by sociologists between privileged and underprivileged groups in their access to cultural venues, experiences, and criticism, which Beardsley (1973, p. 53) analyses in terms of the “distribution of aesthetic welfare.” Yet this distributive approach to aesthetic injustice is different from the lens of hermeneutical justice, which would analyze such phenomena in terms of not a loss in individual welfare but rather social understanding and agency (Simpson 2017). As Lau (2024, pp. 5–6) emphasizes, having a variety of aesthetic experiences gives us the “aesthetic range and depth [...] to appreciate each other’s aesthetic qualities,” not only “raw physical beauty” but also wit, touch, style, and what Lau terms “moral beauty.”

In line with the idea that there is a close connection between the capacity for aesthetic judgement and interpersonal understanding, Tietje and Cresap (2005, p. 37) argue that “much depends on our ability to make valid aesthetic judgments” about the body. According to these authors, “[t]he most obvious case is sexual attraction”, but also “culture, romance, friendship, familial affiliation, imagination, art and major sectors of the economy are unthinkable without judging by appearances” (Tietje and Cresap 2005, p. 37). While these phenomena are certainly held together by a broadly shared capacity for aesthetic evaluation, it may be possible to observe the social phenomenon of aesthetic evaluation of the body without being afforded the resources to understand it.

While psychiatrists have been slow to recognize the relationship between aesthetic judgement and mental disorder, Oyebode’s (2023, p. 301) authoritative textbook on clinical psychopathology now has a chapter on “abnormalities of aesthetic perception and praxis.” With the perception aspect of his overview being most relevant to bodily aesthetics, Oyebode details the emerging evidence that common mental disorders can disrupt typical aesthetic perception of human and non-human forms. For example, Oyebode (2023, p. 305) notes that across the conditions of obsessive-compulsive disorder, Tourette’s syndrome, and body dysmorphic disorder there is a “heightened aesthetic sensitivity” to symmetry, be it in faces or physical objects. This divergence between a patient’s aesthetic judgements and dominant aesthetic norms may not be inherently problematic, but it could lead to an uneasy sense of discord if the nature and source of the patient’s aesthetic experiences were not clear to them.

Other disorders affecting one’s understanding of the appropriate application of aesthetic norms to bodily appearance are those that target perception of the face more specifically, with prosopagnosia and prosopometamorphopsia being especially relevant. Prosopagnosia (or ‘face-blindness’) is more common and characterized by an inability to recognize previously encountered faces, whereas prosopometamorphopsia (or ‘demon-face syndrome’) causes sufferers to perceive faces in distorted, and sometimes grotesque, ways. Such disorders have been described as impairing social cognition, or “the computations that underlie our ability to conceptualize and reason about the social world” (Duchaine et al. 2009, p. 620), due to the process of face perception’s “huge ramifications for social functioning” (Blom et al. 2014). In the obvious case of sexual attraction, the following remarks from Duchaine convey a sense of the experience of aesthetic bewilderment that can result from disorders of facial perception:

“I remember hearing from people who’ve said things like, ‘you know, my girlfriends and I sit in the bar and men are walking past and they’re talking about which guys are attractive and which guys aren’t and I have no idea what they’re talking about. I just look at the faces and I just don’t pick up any attractiveness signal’” (Brain in a Vat 2024).

It seems clear that a person who is unable to understand others’ aesthetic appraisals of human physical appearance will be at a hermeneutical disadvantage, but is it an unfair disadvantage? For a deficit in understanding the social experience of physical beauty appraisal to constitute a hermeneutical injustice along Fricker’s lines, it needs to be avoidable and allowed to occur, or indeed actively encouraged, due to prejudice. Such prejudice is difficult to demonstrate in the abstract, but aesthetic functioning seems susceptible to forms of the more general dismissal of patients’ concerns as trivial, e.g. via the familiar ‘drama queen’ stereotype (Kidd and Carel 2016).

To make a case for the view that such deficits are *avoidable*, one can point to the availability of psychosocial interventions to help patients with, for example, prosopometamorphopsia or another abnormality of aesthetic perception to make sense of their experiences. For example, Winton-Brown et al. (2023) report on the treatment of a patient with prosopometamorphopsia linked to epileptic seizures and a “vivid forearm tattoo” (p. 1) depicting his facial perceptions. Akin to work in psychiatry that advocates using tattoos as a “valuable window into the psyche” (Roggenkamp et al. 2017, p. 157), Winton-Brown et al. suggest that the tattoo “facilitated a therapeutic dialogue [...] and the reclamation of [the patient’s] identity, despite his illness and the frightening visions that had accompanied it” (2023, p. 2).

Moreover, by expanding its aesthetic categories, society could lessen patients’ confusion about aesthetic assessments of the body, such as by widening the notion of ‘attractiveness’ to include gait, mannerisms, and the like as legitimate bodily beauty-markers. In Duchaine’s example above, one might be able to make sense of friends’ comments on the attractiveness of a passer-by if one’s opinions on these attributes were more readily accepted as instances of aesthetic testimony. Such changes could resemble cases of conceptual innovation already suggested as ameliorating hermeneutical injustice whereby dissenting hermeneutical perspectives are able to shape the development of new conceptual formulations (Goetze 2017). The outcome could be the inclusion of a wider range of embodied experiences in the aesthetic domain, fostering aesthetic hermeneutical *justice* by creating space for individuals to contribute meaningfully to shared understandings of bodily aesthetics.

Abnormalities of aesthetic perception such as prosopometamorphopsia or prosopagnosia are relatively rare, of course, and the aesthetic sensitivity discussed above is limited to specific patient populations; but concerns linked to aesthetic hermeneutical injustice may appear across the healthcare domain. Even patients whose aesthetic processing is typical may find themselves inexplicably struggling to reconcile their picture of beauty with the “hidden aesthetic of medical practice” (Stafford et al. 1989), which privileges conformity over imperfection. As Irvin (2022) argues, people experience their bodies as aesthetically imperfect but make such judgements based on functions that they assign to their bodies, which give rise to “one’s self-conception” (p. 272) in aesthetic terms. To capture a multitude of views on such imperfections, medicine could make use of cultural resources such as the Japanese notions of *wabi-sabi*, which “acclaims beauty in common irregularity,” or *kintsugi*, which “celebrates beauty in visible signs of repair, like scars” (Buetow and Wallis 2017, p. 389). By doing so, the healthcare domain may draw ‘unconventional’ conceptions of beauty into the collective pool of hermeneutic resources with which all patients can align themselves to participate in the construction, comprehension, and application of bodily aesthetic norms.

## Discussion

This paper has identified two new forms of aesthetic injustice that are claimed to extend and deepen our understanding of the ways in which appearance norms and aesthetic capacities are bound up with recognition, autonomy, and dignity in healthcare:

1. Aesthetic testimonial injustice, whereby individuals’ aesthetic testimony about their own bodies is unjustly discredited.
2. Aesthetic hermeneutical injustice, whereby individuals are unfairly disadvantaged in making sense of and communicating their experiences of aesthetic evaluation as it pertains to bodies in general.

Patients’ aesthetic testimony, whether it is about how their prostheses should be designed, how their bodies should be dressed or displayed, or anything else, will have to compete with institutional norms of cleanliness or propriety. The medical model of disease’s emphasis on functionality and standardization can sideline or silence patients’ aesthetic values, thus contributing to testimonial aesthetic injustice, yet healthcare also holds possibilities for resisting these injustices. Institutional frameworks shape the conditions under which aesthetic testimony is heard or silenced, and the idea of aesthetic testimonial injustice could help to guide reforms that embed aesthetic agency and dignity into healthcare practice such that aesthetic testimonies are respected. Concrete experiences of such injustices and the possibilities for their rectification merit exploration in future research, and it seems from this paper that aesthetic testimonial injustice can be a useful lens on forms of self-advocacy that challenge prevailing medical paradigms. That said, respect for aesthetic testimony must be balanced with careful ethical and clinical judgement as there may be situations, such as those involving Body Integrity Identity Disorder, where compliance with requests made on aesthetic grounds would be ethically problematic.

Patients’ aesthetic understanding, meanwhile, can be inhibited by a lack of shared language or interpretive resources for making sense of social experiences that are implicitly or explicitly aesthetic in nature and pertain to the body. When locked out of socially acknowledged frameworks to interpret and express these experiences, individuals may be left in a state of aesthetic bewilderment or disorientation that impairs self-understanding and interpersonal agency, thus amounting to aesthetic hermeneutical injustice. Crucially, though, aesthetic categories are not static; and expanding categories by, for example, recognizing diverse forms of beauty and aesthetic expression may mitigate aesthetic hermeneutical injustice, as might dialogue with patients experiencing unusual aesthetic perceptions. Just as epistemic injustice scholars argue that broadening institutions’ interpretive frameworks enables patients to articulate experiences generally excluded from medicine (Carel and Kidd 2023), future research on aesthetic hermeneutical injustice could explore how aesthetic understanding may be afforded by similar means in healthcare.

To preempt an objection that may be levelled against my discussion of aesthetic hermeneutical injustice, it should be noted that to call the aesthetic evaluation of appearance a kind of 'social experience’ is not to suggest that (human physical) beauty is entirely socially constructed. As Minerva (2016, pp. 184–5) explains, there is a basic polarization in debates on beauty between the “evolutionary-essentialist” and “constructionist-social” views that see beauty as, respectively, “a biological adaptation” and “a social construct.” Psychological studies certainly suggest that perceptions of human appearance are largely shared across the species; for example, “preferences for facial averageness and symmetry are not restricted to Western cultures, consistent with the view that they are biologically based” (Rhodes et al. 2001, p. 611). Yet even these aesthetic preferences pertaining to the body are mediated and communicated to individuals through “social cues and injunctive norms” (Lin and McFerran 2016, p. 79), and deviation from the norms has a social cost, as discussions of lookism illustrate. As individuals learn which bodily features are emphasized, how deviations are judged, and how bodies are celebrated or stigmatized, aesthetic experience comes to provide fertile ground for injustice.

This paper’s discussion of aesthetic concerns in healthcare resonates with important but neglected currents within the field of bioethics. As Macneill (2017) argues, bioethics has traditionally operationalized principles such as autonomy, justice, and beneficence through abstract, procedural, or rationalist frameworks that leave little room for the expressive, affective, and sensory dimensions of life. Within Carlin’s (2019) novel approach of “pastoral aesthetics,” the traditional principles of bioethics are correlated with images of care derived from pastoral theology, with justice being correlated with “the living human web” (p. 113) as an image contextualizing human suffering. Yet the conception of justice itself that prevails in bioethics has been unaffected by philosophical discussions about forms of exclusion or marginalization directly pertaining to aesthetic judgement. The concepts of aesthetic testimonial injustice and aesthetic hermeneutical injustice are offered here partly in the hope that they may help to fill this gap in bioethical analysis by identifying further forms of justice linked to aesthetic values and perceptions relating to the body in the context of healthcare.

## Conclusion

In proposing the concepts of aesthetic testimonial injustice and aesthetic hermeneutical injustice, this paper has aimed to show that aesthetic perception and expression are not peripheral to healthcare but central to how patients are heard, understood, and respected. These forms of injustice draw specific attention to the harms that can arise when patients’ aesthetic self-understandings are discredited or rendered unintelligible within institutional settings. By bridging debates on lookism and aesthetic agency and situating them within the framework of epistemic injustice, I hope to have highlighted a critical yet overlooked dimension of justice in healthcare that concerns how bodies are seen, judged, and made meaningful. Of course, it is possible that there are theoretical resources other than those of epistemic justice that are better suited to addressing such phenomena, yet in that case, this paper should be taken as a stimulus for other scholars to make such proposals. By recognizing such a plurality of forms and conceptions of aesthetic injustice, scholars can enrich the conceptual landscape of fields such as bioethics in ways that elucidate how healthcare can affirm patients’ dignity as embodied aesthetic subjects.
